# Egg white hydrolyzate reduces mental fatigue: randomized, double-blind, controlled study

**DOI:** 10.1186/s13104-020-05288-8

**Published:** 2020-09-18

**Authors:** Mariko Oe, Hisae Sakamoto, Hiroshi Nishiyama, Ryou Sasahara, Yasunobu Masuda, Mizuho Adachi, Tetsunari Nishiyama

**Affiliations:** 1 R&D Division, Kewpie Corporation, 2-5-7, Sengawa-cho, Chofu-shi, Tokyo, Japan; 2 Nippon Sport Science University, 7-1-1 Fukazawa, Setagaya-ku, Tokyo, 158-8508 Japan

**Keywords:** Egg white peptide, Anti-fatigue, Antioxidative activity, Dietary supplement, Beverage

## Abstract

**Objectives:**

This study aimed to show that ingesting egg white hydrolyzate (EWH) could improve antioxidant capacity and reduce mental fatigue. Two clinical trials were conducted to examine the antioxidant capacity and the fatigue reduction function of EWH. In Study 1, 19 athlete students were received a single dose of EWH (5 g/day) or placebo. In Study 2, 74 athlete students ingested EWH (5 g/day) or placebo before training for 2 weeks.

**Results:**

Single dose of EWH significantly increased the antioxidant ability compared with the placebo group (p < 0.05), and there was no significant difference between the groups in the oxidative stress test results on Study 1. Two-week intake of EWH significantly decreased mental fatigue compared with the placebo (p < 0.05). This study showed that ingesting EWH improved antioxidant capacity with a single dose and reduced mental fatigue after 2 weeks of ingestion.

*Trial Registration* Japan Medical Association Center for Clinical Trials identifier; JMA-IIA00395 (Study1) and JMA-IIA00396 (Study2), both trials were retrospectively registered on 26 October, 2018.

## Introduction

Proteins, peptides, and amino acids are common sports supplements. In particular, peptides are more absorbable than amino acids of identical composition [1]. Egg white peptide (EWP) has various physiological functions, such as angiotensin-converting enzyme-inhibitory, antioxidant, and anti-inflammatory properties [2]. Among these functions of EWPs, the antioxidant property is the one that is most associated to fatigue reduction after exercise.

Although the anti-fatigue effect of EWP has been reported, the subjects were not athlete and antioxidant activity in vivo has not been studied [3, 4]. Therefore, two clinical studies were conducted to investigate the antioxidant power in the blood and fatigue reduction function after exercise by ingestion of EWH. Study 1 examined the fluctuation of fatigue and antioxidant activity in vivo after a single ingestion of EWH, measured after exercise. Study 2 examined the function of reducing fatigue after exercise with EWH ingestion for 2 weeks.

## Materials and methods

### Ethical consideration

This study was conducted in accordance with the principles of the Declaration of Helsinki and was approved by the Ethical Review Board of Nippon Sport Science University. The approval number is 017-H111 (Study 1) and 017-H112 (Study 2). This study is registered at the Japan Medical Association Center for Clinical Trials (JMACCT) with the clinical trial number JMA-IIA00395 (Study 1) and JMA-IIA00396 (Study 2).

#### Study 1

##### Test food

The EWH used in this study was manufactured by the Kewpie Corporation (Tokyo, Japan) and had an average molecular weight of approximately 700 [5]. A jelly beverage containing EWH (5 g/day; Fujisho Foods Co., Chiba, Japan) was used as the test food for the EWH group, and a jelly beverage without EWH was used for the placebo group (Table 1). The test food was a 130-g drink pack, and the participants ingested one drink pack 1 h before exercise.

**Table 1:** Ingredients and nutritional values of the test food

Ingredients	Nutrition facts(/d)		
		Placebo	EWH
Isomerized sugar, granulated sugar, polysaccharide thickener, citric acid, concentrated grapefruit juice, sweetener, flavor (EWH 5 g/d)	Calories	113 kcal	133 kcal
	Total fat	< 0.1 g	< 0.1 g
	Sodium	209 mg	159 mg
	Total carbohydrate	28 g	29 g
	Protein	0 g	4 g

##### Participants

The participants were 19 athlete students belonging to the bicycle racing club of Nippon Sport Science University. Inclusion exclusion criteria was showed Additional file 1. The sample size was determined from previous studies (Sugiyama et al. unpublished data). For a type 1 risk α of 0.05 and power (1-β) of 80%, the total sample size required was more than 30. The Sample size calculation software was used Cancer research and biostatistics statistical tools (https://stattools.crab.org/). Participants provided informed consent and were randomly allocated in blocks of 4 into the placebo group or EWH (5 g/day) group by using block randomization. Block randomization is a commonly used technique in clinical trial design to reduce bias and achieve balance in the allocation of participants to treatment arms, especially when the sample size is small. Participants were enrolled in the study by the staff not involved in the randomization process. The randomized code was kept in opaque sealed envelope. Both the participants and the investigators were blinded until completion of the trial. The allocation information was concealed in opaque and sealed envelopes by the statistician who was blinded to the group allocation.

##### Clinical trial design

The double-blind, placebo-controlled, randomized, two-period, single-dose, crossover study was conducted at Yokohama Kenshidai Campus, Nippon Sport Science University (Kanagawa, Japan). The participants, care providers and those assessing outcomes were blinded after assignment to interventions. The athletes ingested the placebo or the EWH food 1 h before exercise. The exercise was incremental for 15 min (3 min, five stages) with ergometers (POWERMAX-VIII, Konami Sports Life Co., Ltd., Kanagawa, Japan). The first stage started with 1.5 watts for males and 1 W for females. The exercise intensity was set for each athlete, and it was assigned to each stage so that 95% VO_2_ max was reached at the fifth stage. The 95% VO_2_ max was determined from the records of VO_2_ max test on December 2017. Oxidative stress (diacron-reactive oxygen metabolites; d-ROMs), antioxidant force (biological antioxidant potential; BAP), and fatigue (Borg Rating of Perceived Exertion Scale [6]; Borg RPE Scale, VAS; Visual Analog Scale) were measured before and after exercise. The VAS was a line of 100 mm, with 0 at the left end of the line representing the best condition with no fatigue and 100 at the right end of the line being unbearable fatigue. D-ROMs and BAP were measured with Free Carrio Duo (Wismerll Co., Ltd., Tokyo, Japan). The study was conducted from January 25th, 2018 to February 9th, 2018.

##### Statistical analysis

All values obtained are expressed as mean ± SE. All statistical analyses were performed using IBM SPSS Statistics 20.0 (IBM Japan, Ltd., Tokyo, Japan). A p value of < 0.05 was considered statistically significant. After confirming normal distribution by the Kolmogorov–Smirnov test, paired t-test was used to compare before and after exercise, and independent t-test was used to compare placebo and EWH group for d-ROMs, BAP. The comparison between groups of Borg RPE Scale and VAS was performed by Mann–Whitney U test, and the comparison between before and after exercise was Wilcoxon signed rank sum test.

#### Study 2

##### Test food

The test food of Study 2 was the same as that of Study 1 (Table 1).

##### Participants

The participants were 74 athlete students belonging to the canoe, triathlon, and weightlifting clubs of the Nippon Sport Science University. Inclusion exclusion criteria was same as Study 1 (Additional file 1). The sample size was determined from previous studies (K. Sugiyama et al. unpublished data). For a type 1 risk α of 0.05 and power (1-β) of 80%, the total sample size required was more than 15. The Sample size calculation software was used Cancer research and biostatistics statistical tools (https://stattools.crab.org/). Participants provided informed consent and were randomly allocated into the placebo group or EWH (5 g/day) group. Randomization was performed in the same way as Study 1.

##### Clinical trial design

The randomized, placebo-controlled, double-blind, parallel-group comparison study was conducted at the Tokyo Setagaya Campus, Nippon Sport Science University (Tokyo, Japan). The participants, care providers and those assessing outcomes were blinded after assignment to interventions.

The participants continuously ingested the placebo or the EWH food 1 h before exercise daily over a two-week period. The exercise performed was regular training at each club. Fatigue was evaluated by Chalder’s fatigue scale (CFS) [7] and VAS prior to ingestion, and at 1 and 2 weeks after ingestion. The VAS was the same scale used for Study 1. The CFS was a subjective evaluation of 0–3 ranks each questionnaire related to chronic fatigue, with rank 0 representing the best condition with no fatigue and rank 3 representing unbearable fatigue.

CFS was evaluated before exercise and VAS was rated before and after exercise. Subgroup analyses were performed for each club separately. The study was conducted from February 19th to March 26th, 2018.

##### Statistical analysis

The comparison between groups of CFS and VAS was performed by Mann–Whitney U test, and the comparison between before and after exercise was Wilcoxon signed rank sum test.

## Results

### Study 1

#### Participant background

Nineteen participants who understood the contents of this study and provided consent participated in the study. One athlete withdrew from the study due to personal reasons, with 18 participants remaining (mean age 20 ± 0.2, Additional file 2). Three unhealthy athletes were excluded because of the study aimed at assessing healthy individuals. Thus the analysis was per protocol set. Since this study design was crossover test, 30 data can be obtained from 15 participants, but eight control group and 11 EWH group blood data were also excluded because unable to be analyzed due to hemolysis or insufficient collection volume in blood tests. Finally, seven control group and 11 EWH group data were analyzed on ROMs and BAP. There were no adverse events related to EWP ingestion.

#### Oxidative stress and antioxidant power

There was no significant difference before and after exercise in d-ROMs, but the BAP values for both groups significantly increased after exercise compared with before exercise (p < 0.05).

**Fig. 1:**
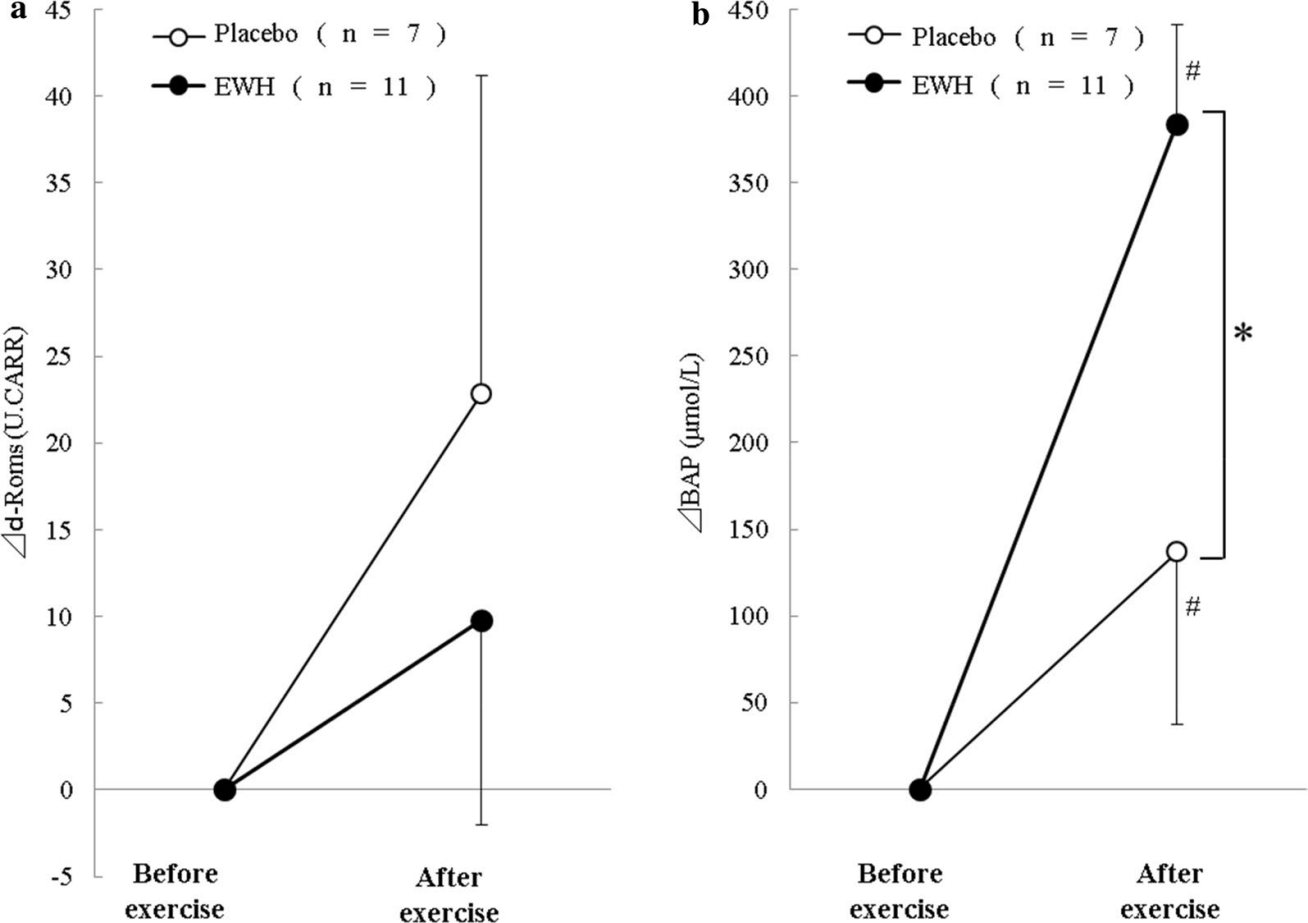
Amount of change before and after exercise in d-ROMs (**a**) and BAP (**b**) values following ingestion of placebo or EWH. Data are presented as mean ± SE, *p < 0.05 vs. placebo group, #p < 0.05 vs. before exercise

Figure 1 shows the changes in d-ROMs and BAP values after exercise versus before exercise. Although there was no significant difference in d-ROMs, the BAP values in the EWH group were significantly higher after exercise than in the control group (p < 0.05).

#### Fatigue questionnaire

There was no significant difference between groups in Borg RPE Scale and VAS values.

### Study 2

#### Participant background

A total of 74 participants (mean age 19.8 ± 0.1) who understood the contents of this study and provided consent participated in the study. There were no participant withdrawals during the study period. There were no adverse events related to daily EWP ingestion. The analysis was full analysis set (Additional file 3).

#### Fatigue questionnaire

**Fig. 2:**
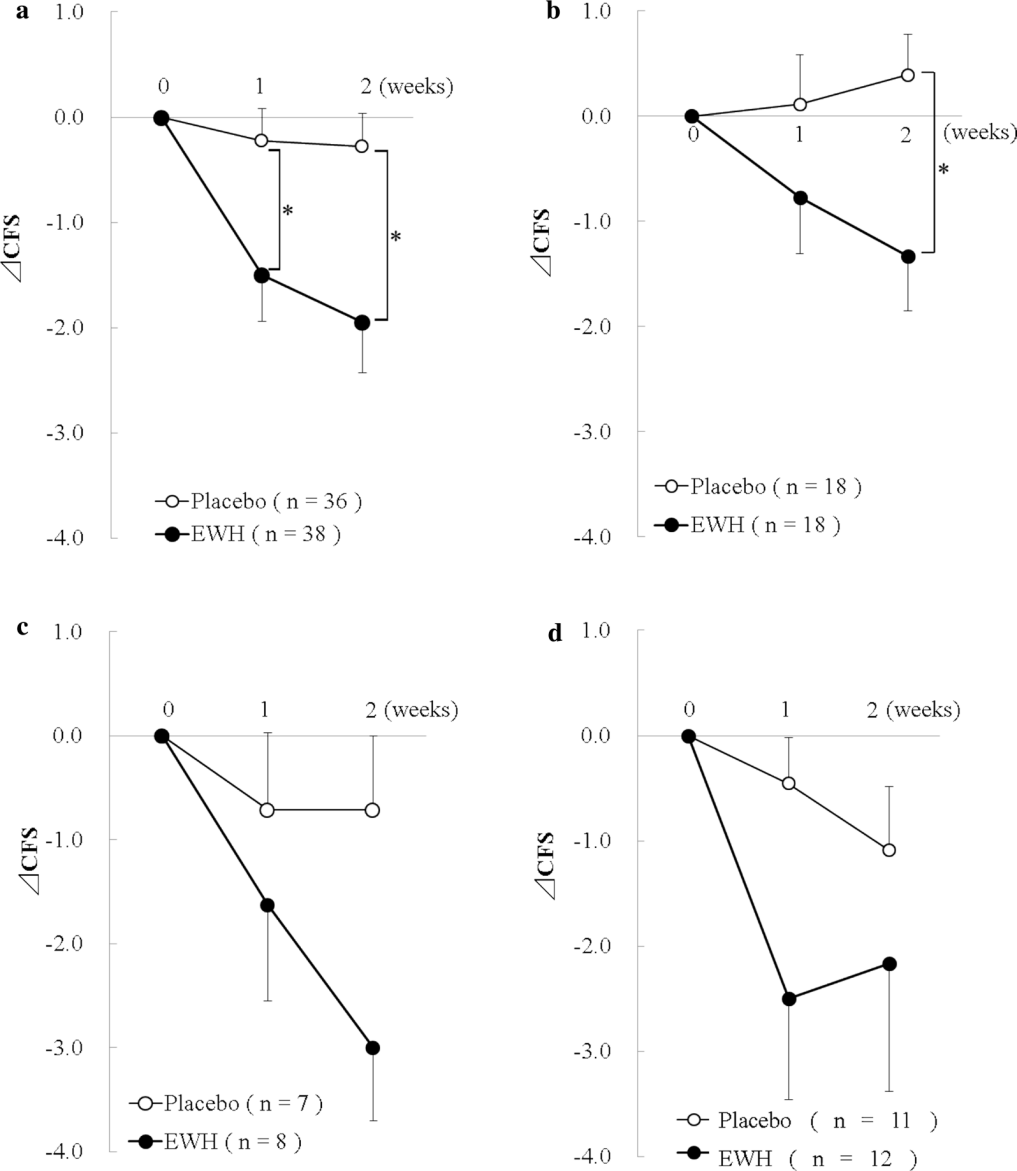
Changes in CFS values for mental fatigue following ingestion of placebo or EWH.** a**, all participants;** b**, canoe club;** c**, triathlon club;** d**, weightlifting club; data are presented as mean ± SE, *p < 0.05 vs placebo

There was no significant difference in CFS values for total fatigue and physical fatigue between the groups. Figure 2 shows the change from baseline CFS values in mental fatigue. Fatigue in the EWH group significantly decreased in total participants and in canoe club participants (p < 0.05). There was a downward trend but no significant toward fatigue in triathlon club and weightlifting club participants in the EWH group compared with those in the placebo group (p < 0.01). Regarding the VAS values, no significant differences were observed for all clubs.

## Discussion

No significant differences in d-ROMs (blood oxidative stress index) were found between the EWP and placebo groups. However, BAP (blood antioxidative activity index) were significantly higher (p<0.05) in the EWP group than in the placebo group. That indicates that compared with placebo intake, EWP intake before exercise resulted in higher antioxidative activity in blood. Antioxidizing enzymes that decompose active oxygen exist in the muscle fiber cytoplasm and mitochondrial matrix. The function of these antioxidant enzymes is complemented by nonenzymatic antioxidants such as peptides [8]. The results of Study 1 show that EWH, a nonenzymatic antioxidant, rapidly enhances the activity of antioxidant enzymes and enhances the in vivo antioxidant capacity in a single intake.

In Study 2, the EWH group had significantly reduced CFS values for mental fatigue compared to the control group at 1 and 2 weeks after ingestion (p < 0.05). CFS is a fatigue index recommended by the Centers for Disease Control and Prevention as a reliable indicator [9]. Ingestion of egg white protein increases the serum amino acid levels [10], intake of egg-derived amino acids had a beneficial effect on mental functions such as emotion and cognition [11].

From these facts, it is considered that the peptide or amino acid derived from EWH is transferred to the blood and acts to reduce mental fatigue. The role of EWP in promoting nitric oxide–related signaling in vivo is considered to be one of the mechanisms of action [12]. The function of EWP is to promote signal transduction related to nitric oxide (NO) in vivo. NO and reactive oxygen (ROS) react in vivo and become active NO species (RNOS), which results in adjustments to the autonomic nervous system [12, 13]. Study 2 showed that these processes were estimated to require continuous intake of EWH for 1 to 2 weeks until a threshold is reached, resulting in recognition of a reduction in mental fatigue. The function of alleviating mental fatigue after exercise due to EWH intake confirmed by this study is a unique feature not confirmed in other food materials.

## Conclusion

This study showed that a single intake of 5 g/day of EWH increased antioxidant power in the blood, and 2 weeks of continuous intake of EWH decreased mental fatigue.

## Limitations

In this study, EHW’s functionality for physical fatigue was not confirmed. The turnover of human muscle proteins is about 180 days in half-life [14]. Further EWH functional studies to reduce physical fatigue need to be conducted long-term over several months based on the muscle protein metabolic cycle. Because this study targeted athlete students, whether EWH intake will have the same impact of anti-fatigue function and antioxidant capacity in the general population with no strong activity intensity is unknown. CFS is a scale for chronic fatigue, that is required the review of applicability for healthy athletes. The decrease in the number of blood analyses is a severe limitation on this study. Further research is required including the statistical planning.

## Supplementary information

**Additional file 1.** Inclusion exclusion criteria.

**Additional file 2.** Participant characteristics in Study 1. EP, ingested EWH in the first period placebo in the second period; PE, ingested placebo in the first period and EWH in the second period.

**Additional file 3.** Participant characteristics in Study 2.

## Data Availability

Not applicable.

## Abbreviations

EWP: Egg white peptide
d-ROMs: Diacron-reactive oxygen metabolites
BAP: Biological antioxidant potential
Borg RPE Scale: Borg Rating of Perceived Exertion Scale
CFS: Chalder’s Fatigue Scale

## References

[1] Adibi SA, Mercer DW (1973). Protein digestion in human intestine as reflected in luminal, mucosal, and plasma amino acid concentrations after meals. J Clin Invest..

[2] Liu YF, Oey I, Bremer F, Carne A, Silcock P (2017). Bioactive peptides derived from egg proteins: a review. Crit Rev Food Sci Nutr.

[3] Sun S, Niu H, Yang T, Lin Q, Luo F, Ma M (2014). Antioxidant and anti-fatigue activities of egg white peptides prepared by pepsin digestion. J Sci Food Agric.

[4] Sugiyama K, Kawada C, Uno S, Yoshida H, Kishimoto Y, Taguchi C, Daigo E (2016). The effects of egg white peptides ingestion for long distance runners on antifatigue in endurance training. Res Q Athlet.

[5] Watabe K (2013). Function of egg white peptide “Peptifine”. Jpn Food Sci.

[6] Borg GA (1982). Psychophysical bases of perceived exertion. Med Sci Sports Exerc.

[7] Chalder T, Berelowitz G, Pawlikowska T, Watts L, Wessely S, Wright D, Wallace EP (1993). Development of a fatigue scale. J Psychosom Res.

[8] Ferreira LF, Reid MB (2008). Muscle-derived ROS and thiol regulation in muscle fatigue. Free Radic Biol Med..

[9] Reeves WC, Lloyd A, Vernon SD, Klimas N, Jason LA, Bleijenberg G, Evengard B, White PD, Nisenbaum R, Unger ER (2003). Identification of ambiguities in the 1994 chronic fatigue syndrome research case definition and recommendations for resolution. BMC Health Serv Res.

[10] Hida A, Hasegawa Y, Mekata Y, Usuda M, Masuda Y, Kawano H, Kawano Y (2012). Effects of egg white protein supplementation on muscle strength and serum free amino acid concentrations. Nutrients..

[11] Mohajeri MH, Wittwer J, Vargas K, Hogan E, Holmes A, Rogers PJ, Goralczyk R, Gibson EL (2015). Chronic treatment with a tryptophan-rich protein hydrolysate improves emotional processing, mental energy levels and reaction time in middle-aged women. Br J Nutr.

[12] Feelisch M (2007). Nitrated cyclic GMP as a new cellular signal. Nat Chem Biol.

[13] Sawa T, Zaki MH, Okamoto T, Sawa T, Zaki MH, Okamoto T (2007). Protein S-guanylation by the biological signal 8-nitroguanosine 3′,5′-cyclic monophosphate. Nat Chem Biol.

[14] Toyosawa I, Yoshioka K, Abe A (2008). Familiar food science.

