# Supplementary figures and images for: Egg white hydrolyzate reduces mental fatigue: randomized, double-blind, controlled study
Source: BMC Res Notes. 2020 Sep 18;13:443. doi: 10.1186/s13104-020-05288-8 (PMC7501625; doi:10.1186/s13104-020-05288-8)

## Slide 1
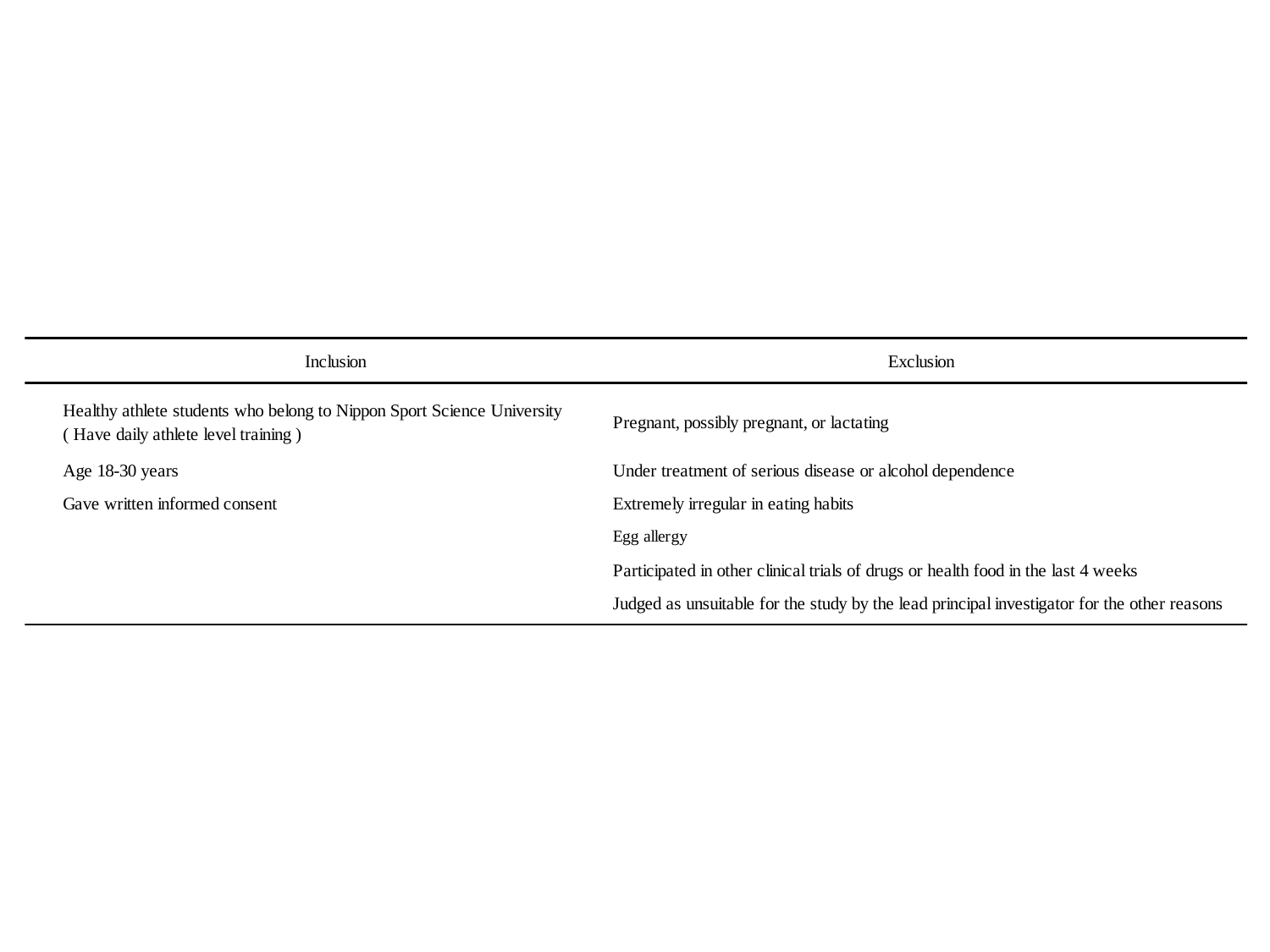

Supplement: Supplementary file 1 — Additional file 1. Inclusion exclusion criteria. [file 13104_2020_5288_MOESM1_ESM.pptx]

## Slide 1
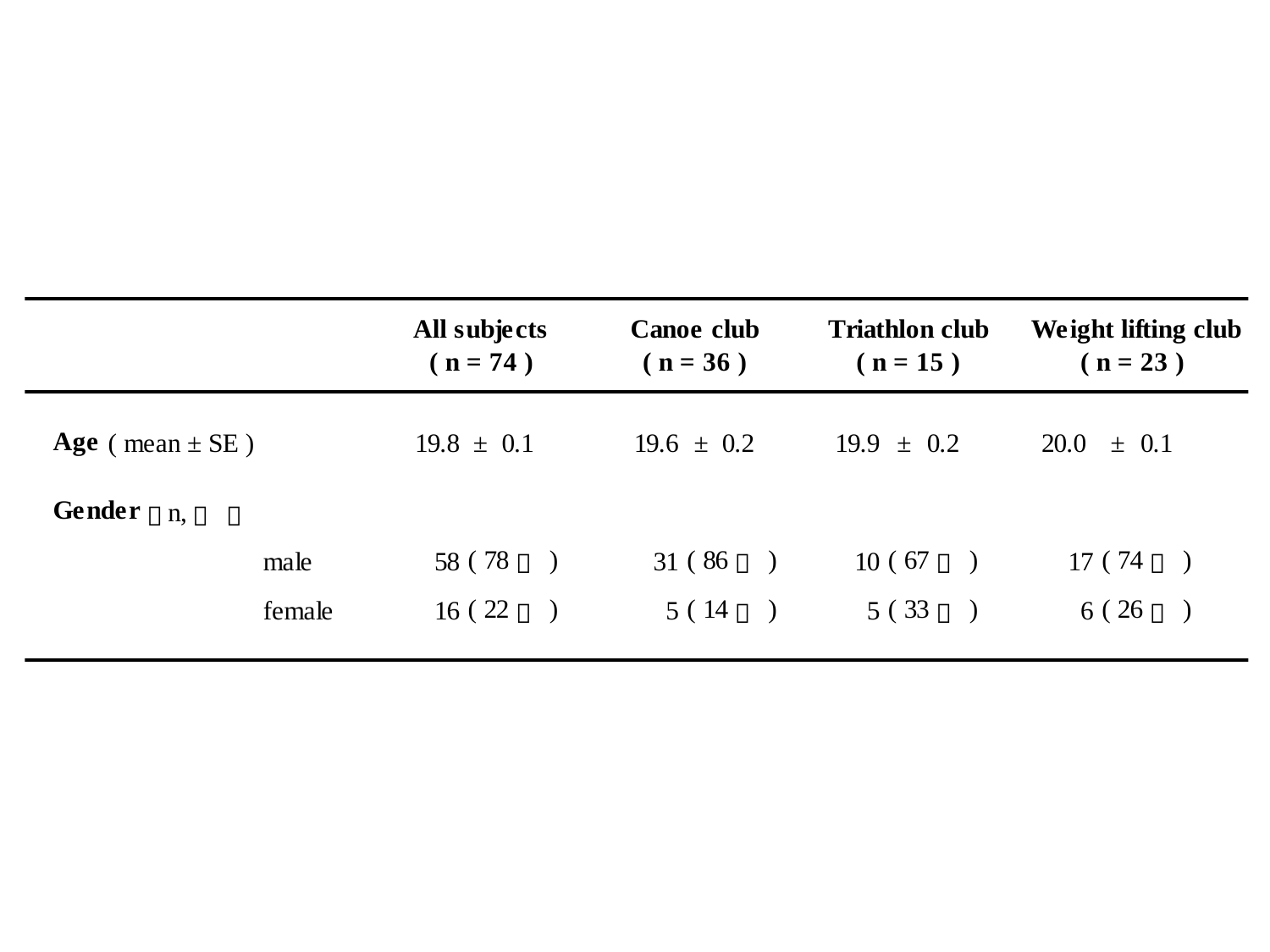

Supplement: Supplementary file 3 — Additional file 3. Participant characteristics in Study 2. [file 13104_2020_5288_MOESM3_ESM.pptx]
