# Supplementary material for: Egg white hydrolyzate reduces mental fatigue: randomized, double-blind, controlled study
Source: BMC Res Notes. 2020 Sep 18;13:443. doi: 10.1186/s13104-020-05288-8 (PMC7501625; doi:10.1186/s13104-020-05288-8)
Supplement: Supplementary file 2 — Additional file 2. Participant characteristics in Study 1. EP, ingested EWH in the first period placebo in the second period; PE, ingested placebo in the first period and EWH in the second period. [file 13104_2020_5288_MOESM2_ESM.pptx]

## Slide 1
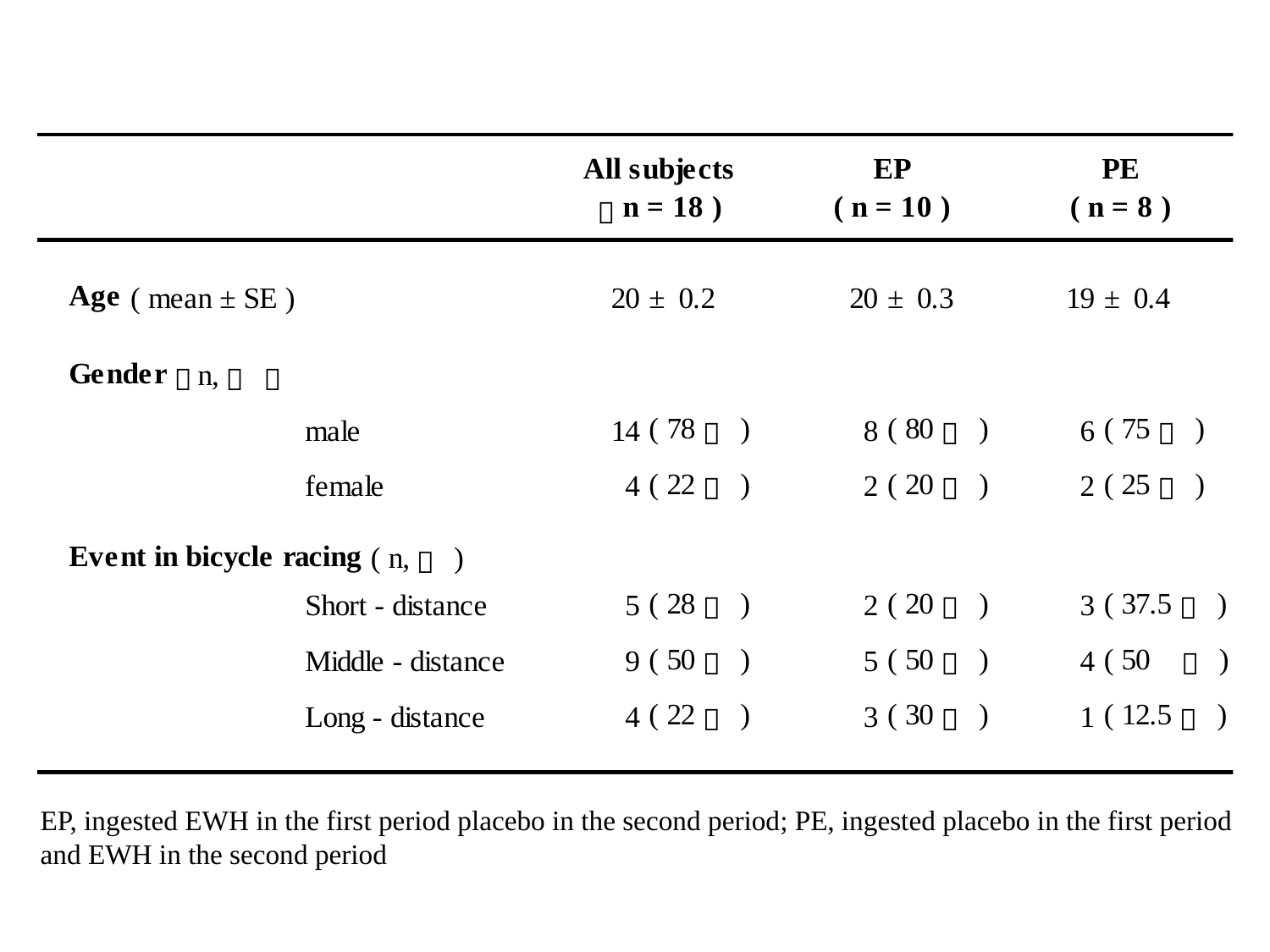

EP, ingested EWH in the first period placebo in the second period; PE, ingested placebo in the first period and EWH in the second period
